# Prevalence and Determinants of Metabolic Health in Subjects with Obesity in Chinese Population

**DOI:** 10.3390/ijerph121113662

**Published:** 2015-10-28

**Authors:** Ruizhi Zheng, Min Yang, Yuqian Bao, Hong Li, Zhongyan Shan, Bo Zhang, Juan Liu, Qinguo Lv, Ou Wu, Yimin Zhu, Maode Lai

**Affiliations:** 1 Department of Epidemiology & Biostatistics, School of Public Health, Zhejiang University, Hangzhou 310058, China; E-Mail: canprezrz@126.com; 2 Department of Nutrition, School of Public Health, Zhejiang University, Hangzhou 310058, China; E-Mail: ymin36@zju.edu.cn; 3 Shanghai Diabetes Institute, Shanghai Jiao Tong University Affiliated Sixth People’s Hospital, Shanghai 200233, China; E-Mail: byq522@163.com; 4 Department of Endocrinology, Sir Run Run Shaw Hospital, Affiliated to School of Medicine, Zhejiang University, Hangzhou 310016, Zhejiang, China; E-Mail: lihongheyi@126.com; 5 Department of Endocrinology and Metabolism, The First Affiliated Hospital, China Medical University, Beier Road No. 92, Shenyang 110001, China; E-Mail: shanzhongyan@medmail.com.cn; 6 Department of Endocrinology, China-Japan Friendship Hospital, Beijing 100029, China; E-Mail: drbozhang@yahoo.com; 7 Department of Endocrinology and Diabetes Center, The First Affiliated Hospital of Sun Yat-sen University, Guangzhou 510080, China; E-Mail: Liujuan8093@126.com; 8 Department of Endocrinology and Metabolism, West China Hospital, Sichuan University, Chengdu 610041, Sichuan, China; E-Mail: lqg3713@163.com; 9 Department of Chronic and Noncommunicable Disease Control and Prevention, Hangzhou Center for Disease Control and Prevention, Hangzhou 310021, China; E-Mail: wuou1@163.com; 10 Department of Pathology, Zhejiang University School of Medicine, Hangzhou 310058, China

**Keywords:** metabolic, prevalence, obesity, dietary, public health

## Abstract

*Background*: The study was to investigate the prevalence of metabolic health in subjects with obesity in the Chinese population and to identify the determinants related to metabolic abnormality in obese individuals. *Methods*: 5013 subjects were recruited from seven provincial capitals in China. The obesity and metabolic status were classified based on body mass index (BMI) and the number of abnormalities in common components of metabolic syndrome. *Results*: 27.9% of individuals with obesity were metabolically healthy. The prevalence of the metabolically healthy obese (MHO) phenotype was significantly decreased with age in women (*p*_trend_ < 0.001), but not significantly in men (*p*_trend_ = 0.349). Central obesity (odds ratio [OR] = 4.07, 95% confidence interval [CI] = 1.93–8.59), longer sedentary time (OR = 1.97, 95%CI = 1.27–3.06), and with a family history of obesity related diseases (hypertension, diabetes, dyslipidemia) (OR = 1.85, 95%CI = 1.26–2.71) were significantly associated with having metabolic abnormality in obese individuals. Higher levels of physical activity and more fruit/vegetable intake had decreased ORs of 0.67 (95%CI = 0.45–0.98) and 0.44 (95%CI = 0.28–0.70), respectively. *Conclusion*: 27.9% of obese participants are in metabolic health. Central obesity, physical activity, sedentary time, fruits/vegetables intake and family history of diseases are the determinants associated with metabolic status in obesity.

## 1. Introduction

Obesity has become one of the most prevalent metabolic diseases worldwide. Obesity is a key risk factor for dyslipidemia, hypertension, type 2 diabetes, cardiovascular diseases (CVD), certain types of cancer [1], and higher mortality in related chronic diseases [2]. However, obesity as defined by the body mass index (BMI) is a heterogeneous condition, and around 30% of obese individuals named metabolically healthy obese (MHO), despite having excessive body fat, display favorable metabolic profiles characterized by high levels of insulin sensitivity, no hypertension, as well as favorable lipid, inflammation, hormonal, liver enzyme and immune profiles [3,4,5,6].

Previous studies indicated that the MHO population is protected from the development of cardiometabolic disease compared with metabolically abnormal obese individuals [7,8]. In addition, treatment of obesity and prevention of obesity-related diseases is an enormous medical and socioeconomic task which will not always be successful. Therefore, it is beneficial to identify potential risk and protective factors associated with metabolic abnormality in obese subjects. Possibly correlations have been reported such as age, gender, ethnicity/race, physical activity, smoking, alcohol intake and dietary, but the results are inconsistent [9,10,11].

Due to rapid transition of dietary behaviors and lifestyles, China is experiencing an epidemic of obesity and metabolic disease in the last few decades [12]. Few studies had examined the characteristics of the MHO phenotype in the Chinese population. In this regard, we investigated the prevalence and characteristics of MHO individuals in seven capital cities in China, and provided evidence in relation to the role of dietary and lifestyle factors in determining metabolic status in obese individuals.

## 2. Methods

### 2.1. Study Population

**Figure 1:**
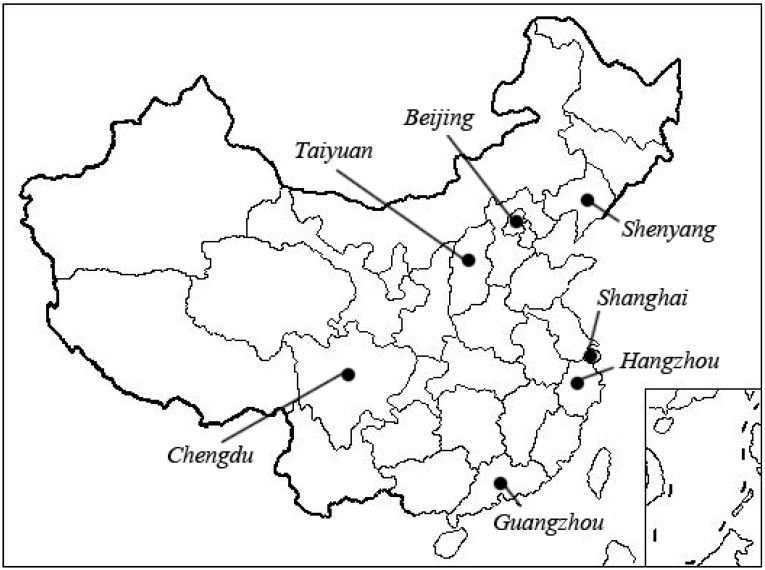
Locations of the recruitment in China.

The subjects in this study were recruited from our previous cross-sectional investigation on metabolic syndrome carried out in China in 2010 (Cross-Sectional Investigation on Metabolic Syndrome, CSIMS2010). In CSIMS2010, the subjects, aged over 20 years old, were randomly sampled from seven geographically representative areas in China (Shanghai and Hangzhou for eastern China, Beijing for northern China, Shenyang for northeast China, and Taiyuan for central China, Chengdu for southwest China and Guangzhou for southern China) (Figure 1). In each city, one or two communities were selected based on representatives in socioeconomic levels, distributions of age and gender, lifestyle and dietary behaviors with general population. All of the selected communities had not been intervened in for chronic diseases such as metabolic syndrome, diabetes, and cardiovascular diseases. This study was conducted according to the guidelines laid down in the Declaration of Helsinki and all procedures involving human subjects were approved by the institutional review board at the Zhejiang University, Zhejiang, China. Written informed consent was obtained from all participants.

The inclusion criteria of the subjects were as follows: 1 age over 20; 2. living in local community more than two years; 3. Han ethnicity with no restriction on gender. The subjects were excluded if they had severe chronic diseases such as cancer, coronary heart disease, stroke, chronic cirrhosis, hyperthyroidism or hypothyroidism. We also excluded patients with percutaneous coronary intervention, corticosteroid therapy and prior coronary arterial bypass surgery, as well as those who had taken drugs for more than six months or within the previous 12 months that could potentially affect lipid metabolism. After excluding 46 subjects with missing values in questionnaire and medical records, there remained an analytic sample of 5013 participants.

### 2.2. Anthropometric Measurement

Anthropometric indices, including weight, height, waist circumference, systolic blood pressure (SBP), and diastolic blood pressure (DBP) were measured by well-trained investigators, following a standard protocol. Height and weight were measured using a scale, with the subjects wearing light clothing and without shoes. Waist circumference (WC) was measured at the midpoint between the iliac crest and lowest rib. Blood pressure was measured in a sitting position with a mercury sphygmomanometer. SBP and DBP were reported as the average of three repeat measurements with 30-s intervals.

### 2.3. Biochemical Determination

After a 12-h overnight fast, whole blood and serum samples were collected for each subject. Biochemical variables including, triglycerides (TG), total cholesterol (TC), high-density lipoprotein cholesterol (HDL-C) and low density lipoprotein cholesterol (LDL-C) were determined using biochemical auto-analyzers (Hitachi 7060, Tokyo, Japan). Fasting plasma glucose (FPG) was analyzed with a glucose oxidase method with the Beckman Glucose Analyzer (Beckman Instruments, Irvine, CA, USA). A 30 min and 2 h oral glucose tolerance test (OGTT) was performed as a regular procedure for the subjects, except for the previously diagnosed diabetes patients.

### 2.4. Epidemiological Investigation

Each subject was interviewed face to face with a structured questionnaire. The information in the questionnaire included demographic data (date of birth, gender, educational level, marital status, *etc.*), smoking and alcohol drinking behaviors, physical activity, dietary and family histories of diseases, *etc.*

Smoking behavior was grouped as current, former, and never. Current smoking was defined as smoking at least one cigarette per day and lasting for one year. Former smoking was defined as stopping smoking for at least one year. Alcohol drinking was classified in three categories as never, moderate and heavy. Heavy drinking was defined as drinking more than three times per week. Moderate drinking was defined as drinking between the levels of heavy and never.

International Physical Activity Questionnaire (IPAQ) (short vision) was used for the assessment of the average amount of time per week engaged in exercise activities [13]. According to the instruction, the energy expended for each activity in metabolic equivalent (MET) minutes per week (MET-m/week) was calculated. The total physical activity cutoff value ≥600 MET-m/week was considered as moderate or high physical activity level. Sedentary hours per week were calculated, participants were classified as short, moderate and long by the interquartile of sedentary hours.

Trained interviewers inquired about dietary intake using the semi-quantitative food frequency questionnaire (FFQ). For each food item, a common serving size of the food or beverage was specified. Participants were asked the frequency of consumption, on average, of this amount during the previous year. Individuals firstly selected from four possible frequency units ranging from “never or less than once per month” to “1 (or 2) or more times per day” and then selected the appropriate portion size. For non-unitary foods, a photo was provided to aid in estimating the four different portions. For each food, an amount was assigned on the basis of the gram weight of the volume for the selected portion-size model. Each food intake was calculated by summing the product of the frequency of intake and the amount consumed at each frequency unit. In addition, participants were classified as more or less based on mean cut-points of food intake. The grains intake contained rice, flour, breads and ready to eat cereals; meat intake contained pork, beef, lamb and poultry.

### 2.5. Definition of the Phenotypes

General obesity was defined by BMI, which was recommended by the Working Group on Obesity in China (WGOC) [14]. Participants were classified as normal weight (BMI 18.5~24.9 kg/m^2^), overweight (BMI 25~27.9 kg/m^2^), and obese (BMI ≥ 28 kg/m^2^) based on standard cutoffs. Central obesity classified subjects with waist circumference ≥90 cm for male, and ≥80 cm for female.

Metabolically abnormal components included: (1). elevated TG (≥1.7 mmol/L); (2). low HDL-C (men < 1.03 mmol/L, women < 1.29 mmol/L); (3). elevated SBP (≥130 mm·Hg) or DBP (≥85 mm·Hg), or use of antihypertensive drug therapy; (4). elevated fasting plasma glucose (≥5.6 mmol/L) or anti-diabetic treatment [15]. Metabolic health was defined as having the number of metabolically abnormal components ≤ 1, metabolic abnormal was defined as having the number of metabolically abnormal components ≥ 2.

The subjects at normal and obese groups were classified into four categories based on metabolic status: metabolically healthy normal weight (MHNW), metabolically abnormal normal weight (MANW), metabolically healthy obesity (MHO) and metabolically abnormal obesity (MAO).

### 2.6. Statistical Analysis

Continuous variables with normal distribution were described as means and standard deviations (SD), whereas continuous variables with skewed distribution were expressed as median (and interquartile range). Categorical variables were expressed as percentages (%) if data was not at a normal distribution. The comparison of clinical and body composition parameters among the four metabolic phenotypes was performed with one-way analysis of variance (ANOVA), and Bonferroni correction was used to control for the inflation of type I error due to multiple comparisons. The Mann–Whitney U test was used to examine the between-group differences, if data was not at a normal distribution. Statistical differences of the MHO prevalence among age groups and different regions were analyzed using the χ^2^ test. Binary logistic regression models were used to estimate odds ratio (ORs) and 95% confidence intervals (CIs) for the associations of sociodemographic and lifestyle variables with metabolic healthy status in obesity. The model was adjusted for age, gender and all other variables. All *p* values were two-tailed. Statistical significance was defined as *p* < 0.05. All the statistical analysis was conducted using PASW Statistics version 20.0 for Windows (SPSS Inc., Chicago, IL, USA).

## 3. Results

Five-thousand and thirteen subjects, aged 35–72, were recruited in this study. Forty-seven percent of the subjects (2355) were men and 53% (2658) were women. The prevalence of general obesity and central obesity was 14.0% and 17.6%, respectively. Table 1 describes the basic demographic and metabolic characteristics among the four phenotypes. MAO displayed significantly higher anthropometric indices of central adiposity such as waist circumference, waist hip rate and waist-to-height ratio (All *p* < 0.05). As expected, MANW and MAO individuals exhibited a significantly worse cardiometabolic profile than MHNW and MHO.

Table 2 shows the prevalence of metabolic health in obese subjects with different age groups. In obese individuals, 27.9% met the criteria of metabolic health. Women (32.9%) had significantly higher prevalence of MHO than that of men (23.4%). The prevalence of MHO was consistently decreased with age in women (*p*_trend_ = 0.001), but not significant in men (*p*_trend_ = 0.349). The subjects in south China had higher prevalence of metabolic health in obesity (36.8%) than that in north (22.3%). There was no significant difference of the prevalence between men (34.9%) and women (38.9%) in south China (*p* = 0.473).

Table 3 presents the prevalence and determinants of metabolic abnormality (MA) in obese individuals. Elevated waist circumference (>100 cm) was significantly associated with increased odds for MA in obesity (OR = 6.01, 95%CI = 1.30–27.79). The obese individuals with longer sedentary time (OR = 1.97, 95%CI = 1.27–3.06) and family history of diseases (hypertension, diabetes and dyslipidemia) (OR = 1.85, 95%CI = 1.26–2.71) were significantly associated with MA. Moderate or long physical activity (OR = 0.67, 95%CI = 0.45–0.98) and more fruits/vegetables intake (OR = 0.44, 95%CI = 0.28–0.70) were related to lower ORs for MA in obesity.

**Table 1:** The demographic and metabolic characteristics in the subjects of metabolically healthy normal weight (MHNW), metabolically abnormal normal weight (MANW), metabolically healthy obese (MHO) and metabolically abnormal obese (MAO).

Variable	MHNW (*n* = 1661)	MANW (*n* = 655)	MHO (*n* = 196)	MAO (*n* = 506)
Men/women (%)	37.30/62.70	47.80/52.20	43.90/56.10	55.70/44.30
Age (years)	51.82 ± 6.91	54.43 ± 6.94 ^#^	52.01 ± 7.62	53.95 ± 7.75 ^†^
WC (cm)	74.37 ± 6.37	79.02 ± 6.57 ^#^	94.14 ± 8.41 ^‡∫^	98.07 ± 8.59 ^†§¶^
WHR	0.83 ± 0.06	0.87 ± 0.06 ^#^	0.91 ± 0.07 ^‡∫^	0.93 ± 0.07 ^†§¶^
WHtR	0.46 ± 0.04	0.48 ± 0.04 ^#^	0.58 ± 0.05 ^‡∫^	0.60 ± 0.05 ^†§¶^
SBP (mm·Hg)	114.12 ± 13.51	127.81 ± 16.33 ^#^	124.02 ± 15.65 ^‡∫^	137.32 ± 15.69 ^†§¶^
DBP (mm·Hg)	75.63 ± 14.32	83.86 ± 18.24 ^#^	80.87 ± 10.16 ^‡∫^	88.56 ± 10.40 ^†§¶^
TC (mmol/L)	5.09 ± 1.03	5.42 ± 1.11 ^#^	5.04 ± 0.90 ^∫^	5.31 ± 1.05 ^†§^
TG (mmol/L)	1.09 ± 0.67	2.33 ± 1.82 ^#^	1.38 ± 0.98 ^‡∫^	2.45 ± 1.81 ^†§^
HDL-C (mmol/L)	1.62 ± 0.39	1.29 ± 0.38 ^#^	1.43 ± 0.47 ^‡∫^	1.20 ± 0.40 ^†§¶^
LDL-C (mmol/L)	2.84 ± 0.91	2.86 ± 0.89	3.03 ± 0.93 ^‡∫^	3.02 ± 0.91 ^†§^
TC/HDL-C ratio	3.27 ± 0.85	4.60 ± 2.50 ^#^	3.67 ± 0.88 ^‡∫^	4.79 ± 2.07 ^†§^
LDL/HDL-C ratio	1.83 ± 0.68	2.38 ± 1.28 ^#^	2.21 ± 0.77 ^‡^	2.65 ± 0.92 ^†§¶^
UA (mmol/L)	266.56 ± 75.31	292.98 ± 82.89 ^#^	304.52 ± 82.96 ^‡^	332.36 ± 90.07 ^†§¶^
ALT (U/L)	16 (13–22)	20 (15–26) ^#^	21 (16–32) ^‡^	26 (19–38) ^†§¶^
AST (U/L)	20 (17–24)	20 (17–25)	21 (18–25)	22 (19–27) ^†§¶^
γ-GT (U/L)	18 (14–25)	22 (16–34) ^#^	25 (16–41) ^‡^	32 (22–46) ^†§¶^
FPG (mmol/L)	4.96 ± 0.82	5.84 ± 1.76 ^#^	5.17 ± 0.83 ^‡∫^	6.29 ± 2.04 ^†§¶^
30 min OGTT glucose (mmol/L)	8.29 ± 2.05	9.75 ± 3.00 ^#^	8.83 ± 2.15 ^‡∫^	10.61 ± 3.22 ^†§¶^
2 h OGTT glucose (mmol/L)	5.90 ± 2.33	8.05 ± 4.29 ^#^	6.82 ± 2.54 ^‡∫^	8.97 ± 4.54 ^†§¶^

Notes: Definition: Normal weight was defined as 18.5 ≤ BMI < 25 kg/m^2^, obesity was defined as BMI ≥ 28 kg/m^2^. Metabolic health was defined as presenting ≤1 of metabolic abnormality. Data are presented as mean ± standard deviation and median (P25–P75). Abbreviation: MHNW, metabolically healthy normal-weight; MANW, metabolically abnormal normal-weight; MHO, metabolically healthy obesity; MAO, metabolically abnormal obesity; WC, waist circumference; WHR, waist hip rate; WHtR, waist-to-height ratio; SBP, systolic blood pressure; DBP, diastolic blood pressure; TC, total cholesterol; TG, triglycerides; HDL-C, high density lipoprotein cholesterol; LDL-C, low density lipoprotein cholesterol; UA, Uric acid; ALT, alanine aminotransferase; AST, aspartate Aminotransferase; γ-GT, γ-glutamyltransferase; FPG, fasting plasma glucose; OGTT, oral glucose tolerance test. ^#^ MHNW *vs.* MANW, *p* < 0.05; ^‡^ MHNW *vs.* MHO, *p* < 0.05; ^§^MHNW *vs.* MAO, *p* < 0.05; ^†^ MHO *vs.* MAO, *p* < 0.05; ^∫^ MHO *vs.* MANW, *p* < 0.05; ^¶^ MANW *vs.* MAO, *p* < 0.05.

**Table 2:** Prevalence of metabolically healthy and abnormal in obese subjects stratified by age and gender.

	All Subjects		Men		Women		*p* Value *
	Healthy	Abnormal	Healthy	Abnormal	Healthy	Abnormal	
Overall	196 (27.9)	506 (72.1)	86 (23.4)	282 (76.4)	110 (32.9)	224 (67.1)	0.005
Age group							
35–	50 (35.0)	93 (65.0)	22 (25.3)	65 (74.7)	28 (50.0)	28 (50.0)	0.002
45–	84 (31.0)	187 (69.0)	34 (26.0)	97 (74.0)	50 (35.7)	90 (64.3)	0.083
55–	52 (21.5)	190 (78.5)	24 (19.4)	101 (80.6)	28 (23.7)	90 (23.7)	0.408
65–	10 (21.7)	36 (78.3)	6 (23.1)	19 (76.9)	4 (20.0)	16 (20.0)	0.802
*p*_trend_	**<0.001**		0.349		**<0.001**		
Region							
North China	96 (22.3)	335 (77.7)	35 (15.8)	186 (84.2)	61 (29.3)	147 (70.7)	**0.001**
South China	100 (36.8)	171 (63.2)	51 (34.9)	96 (65.1)	49 (38.9)	77 (61.1)	0.473
*p* value ^†^	**<0.001**		**<0.001**		**<0.001**		

Notes: Data are presented as the number of metabolic abnormality (%). * Comparison of the prevalence of metabolically healthy obesity between different genders. ^†^ Comparison of the prevalence of metabolically healthy obesity between north and south China.

**Table 3:** Multiple-adjusted odds ratios for metabolically abnormal phenotype associated with demographic and lifestyle factors in obese individuals.

Variables	MAO [N (%)]	MHO [N (%)]	Odds Ratio *	95%CI	*p* Value
Central obesity					
No	14 (2.7)	17 (8.7)	Reference		
Yes	492 (97.3)	179 (91.3)	4.07	1.93–8.59	**<0.001**
WC group (cm)					
<80	4 (0.8)	6 (3.1)	Reference		
80–	53 (10.5)	52 (26.5)	1.74	0.38–8.00	0.360
90–	252 (49.8)	90 (45.9)	4.00	0.89–17.88	0.070
≥100	197 (38.9)	48 (24.5)	6.01	1.30–27.79	**0.022**
*p*_trend_					**<0.001**
Education level					
Primary	87 (17.3)	25 (12.8)	Reference		
Secondary	349 (68.9)	148 (75.5)	0.56	0.31–1.01	0.053
Senior	70 (13.8)	21 (10.7)	0.83	0.38–1.81	0.634
Smoking status					
Never	280 (55.3)	113 (57.7)	Reference		
Former	46 (09.1)	16 (08.2)	0.65	0.30–1.41	0.276
Current	180 (35.6)	67 (34.1)	0.75	0.43–1.30	0.303
Alcohol intake					
Non-drinker	264 (52.2)	106 (54.0)	Reference		
Moderate drinker	139 (27.5)	55 (28.1)	0.84	0.50–1.40	0.493
Heavy drinker	103 (20.4)	35 (17.9)	0.73	0.39–1.36	0.327
Tea intake					
<3/week	181 (38.5)	72 (39.3)	Reference		
3–5/week	124 (26.3)	49 (26.8)	1.04	0.65–1.68	0.865
>5/week	166 (35.2)	62 (33.9)	1.03	0.64–1.66	0.904
*p*_trend_					0.518
Physical activity ^†^					
Low activity	249 (49.2)	108 (55.1)	Reference		
Moderate or high activity	257 (50.8)	88 (44.9)	0.67	0.45–0.98	0.039
Sedentary time ^‡^					
Short	106 (20.9)	61 (31.1)	Reference		
Moderate or long	400 (79.1)	135 (68.9)	1.97	1.27–3.06	0.002
Grain intake ^§^					
Less	255 (50.4)	101 (51.5)	Reference		
More	251 (49.6)	95 (48.5)	1.25	0.83–1.89	0.289
Meat intake ^§^					
Less	345 (68.2)	127 (64.8)	Reference		
More	161 (31.8)	69 (35.2)	0.99	0.67–1.48	0.984
Fish intake ^§^					
Less	400 (79.1)	149 (76.0)	Reference		
More	106 (20.9)	47 (24.0)	0.83	0.56–1.24	0.358
Fruits/vegetables intake ^§^					
Less	199 (39.3)	52 (26.5)	Reference		
More	307 (60.7)	144 (73.5)	0.44	0.28–0.70	0.001
Milk intake ^§^					
Less	283 (55.9)	100 (51.0)	Reference		
More	223 (44.1)	96 (49.0)	0.84	0.60–1.17	0.302
Egg intake					
<1/week	96 (19.0)	40 (20.4)	Reference		
1–3/week	247 (48.8)	87 (44.4)	1.29	0.82–2.02	0.278
>3/week	163 (32.2)	69 (35.2)	1.01	0.63–1.62	0.974
Family history of diseases ^∫^					
No	184 (36.4)	84 (42.9)	Reference		
Yes	322 (63.6)	112 (57.1)	1.85	1.26–2.71	0.002
Family history of cardiovascular disease					
No	327 (64.6)	139 (70.9)	Reference		
Yes	179 (35.4)	57 (29.1)	1.41	0.98–2.02	0.068

Notes: * Each factor is adjusted for gender, age and other factors in the table. ^†^ The total physical activity cutoff value ≥ 600 MET-m/week was considered as moderate or high physical activity level. ^‡^ Sedentary time is classified by the interquartile of participants' sedentary hours per week. ^§^ More and less of the dietary intakes are defined by the mean cut-points of the amount of the dietary intakes per week. ^∫^ Family history diseases include hypertension, diabetes and dyslipidemia. MAO, metabolic abnormal obesity; MHO, metabolically healthy obesity.

## 4. Discussion

Our findings suggest that MHO phenotypes are widespread among Chinese adults. Different lifestyle factors are linked with the presence or absence of the metabolic abnormality in obese individuals. Elevated waist circumference, longer sedentary time, and family history of obesity related diseases appeared to contribute to higher risk of MA, while more fruits/vegetables intake and higher level of physical activity reduced the risk of MA for obese individuals.

According to the available studies, the reported prevalence of MHO phenotypes ranged widely, depending on the characteristics of the subjects and the diagnostic criteria. Kuk *et al.* reported that only 6% of the obese population was insulin sensitive and metabolic syndrome free [16]. In our study, almost one-third of obese subjects were in metabolic health, which was similar to most American and European studies [6,17]. For instance, Wildman *et al.* and Iacobellis *et al.* found a prevalence of 31.0% and 27.5%, respectively, of metabolically healthy subjects among general obese populations [6,18]. In other Asian countries, the prevalence of metabolically healthy people in Chinese obese individuals was lower than that in Japanese (44.1%) and India (47.3%) [19,20].

Most studies demonstrated that the metabolic status deteriorated with age in both genders, independent of weight status and the criteria applied to define metabolic abnormality [5]. It was in partial accordance with our observations. The body composition shifted to more fat and resulted in weight gain culminating in metabolic abnormalities with age [21]. Many studies also attributed the trend to the decreased level of gender hormones in women. A lot of metabolic changes in postmenopausal women were related to the decrease in estrogen secretion and consequent accumulation of abdominal fat [22]. However, the prevalence of MHO in men was not remarkably changed through age in our study. The present investigation discovered that south China had higher prevalence of MHO than that in the north; these results might be associated with higher intake of dietary lipids and alcohol in the northern population, and in addition, the warm temperature in south China provides more suitable conditions for physical activity [23].

In our study, the larger waist circumference presented greater risk of metabolic abnormality. However, several studies also discovered a large proportion of metabolically healthy individuals with central obesity [24]. It was suggested that both BMI and waist circumference might not completely predict the risk of obesity related to cardiometabolic disease. Visceral adiposity was a key risk factor, given its pathogenic consequences in animal models and its role in metabolic dysfunction based on epidemiologic data [25]. Neither BMI nor waist circumference was closely associated with visceral adiposity; in addition, changes in weight were small compared with concomitant changes in visceral or subcutaneous fat [26]. To better understand the MHO phenotype and its prognosis, further research on the stability and change in metabolic condition over time using more accurate measures of adiposity, and considering genetic and environmental factors would be useful.

In our study, higher levels of physical activity decreased the odds of metabolic abnormality for obese individuals. There was strong evidence indicating that higher fitness levels were associated with fewer metabolic complications and lower prevalence of metabolic syndrome at any age and across different weight status [27]. A better fitness level despite being obese is associated with less visceral and intrahepatic fat accumulation, which reduced the risk of developing type 2 diabetes and cardiovascular diseases [28]. However, other studies observed that being physical active only moderately attenuated but did not eliminate the adverse effect of obesity on cardiovascular health [29]. We also found that longer sedentary time contributed to higher risk of metabolic abnormality. Conus *et al.* indicated that the metabolically abnormal women were less aerobically fit, and spent a greater portion of their time watching television [30]. These types of biological attributes and behaviors likely contributed to the positive energy balance that leads to greater adiposity [30]. This was in agreement with the belief that the natural tendency of the deterioration of metabolic status when physical activity was stopped or reduced.

In present study, more fruits/vegetables might be protective against metabolic abnormality for obese individuals. The same result was also obtained by another study, it compared diet composition between MHO and MAO individuals, and only vegetable protein was higher in the MHO subjects [9]. The dietary fibers contained in fruits and vegetables might prohibit intestinal absorption of cholesterol and bile acids, which resulted in a better serum lipid profile [31]. The antioxidants contained in fruits and vegetables also improved the process of inflammation [32].

The current study indicated family history in obesity related diseases (hypertension, diabetes and dyslipidemia) as important risk factors for developing metabolic abnormality. Similar findings had been reported in previous studies. Sangun *et al.* reported that in children and adolescents, metabolic syndrome prevalence was not only significantly higher in children who had a family history of hypertension, but also with heart disease, diabetes and obesity [33]. Future genetic studies were needed to examine the genetic contribution to the MHO phenotype.

### Strengths and Limitations

The strengths of the present study included a representative sample of adult individuals in seven provincial capitals of China. The survey methods had been carefully standardized and abided by international recommendations. The limitations involved in this study were as follows: Firstly, even though the study samples were from multiple regions, they were mainly urban residents. Future studies are needed to cover the entire population. Secondly, the cross-sectional study design limited the ability to make an inference about the causal relationship between dietary and lifestyle factors and MHO phenotype. And the use of self-reported questionnaires was subject to potential inaccuracies, recall and reporting biases. Thirdly, it is also important to note that the obese individuals might underreport their food intake. Under reporting of energy intake was not random, but greater among people who were obese, older compared with younger and women compared with men [34]. The fourth, BMI index was used to classify subjects as obese, it might misclassify the persons with short stature or muscular build. In future work, dual-energy X-ray absorptiometry (DEXA) might be more accurate to measure the body fat. Lastly, the development of metabolic abnormality occurs on a physiological continuum, and there are no unique criteria to define the MHO phenotype; different results might be observed by adopting other criteria to identify MHO. Thus, it might not be reasonable to determine “health” relying on artificial thresholds, and it is likely preferable to conduct longitudinal studies to define the precision and predictive value of the metabolic disorder signals in the MHO phenotype.

## 5 Conclusions and Implications

This cross-sectional study finds that almost one-third of obese subjects are in metabolic health in China. Central obesity, longer sedentary time, and a family history of obesity-related diseases are associated with increased risk of metabolic abnormality, while more physical activity and fruit/vegetable intake decreased the risk.

The findings of this study have demonstrated that obesity is heterogeneous in metabolic status. The prevalence of obesity was higher in city residents, therefore, effective intervention is urgently needed for obesity control and prevention of related diseases. However, current “one size fits all” approaches to tackle obesity are not successful for most people. More targeted interventions according to different obesity phenotypes are needed. A better understanding of the determinants underlying obesity associated metabolic health subtypes will be beneficial to find out the new targeted or customized therapeutic or intervention strategies to attenuate disease development and reduce the significant economic burden of obesity. This study indicates that physical activity and fruits/vegetables intake increase the likelihood of metabolic health in obesity; these findings also provide evidences for the development of obesity control strategies.

## References

[1] Simmonds M., Burch J., Llewellyn A., Griffiths C., Yang H., Owen C., Duffy S., Woolacott N. (2015). The use of measures of obesity in childhood for predicting obesity and the development of obesity-related diseases in adulthood: A systematic review and meta-analysis. Health Technol. Assess..

[2] Adams K.F., Schatzkin A., Harris T.B., Kipnis V., Mouw T., Ballard-Barbash R., Hollenbeck A., Leitzmann M.F. (2006). Overweight, obesity, and mortality in a large prospective cohort of persons 50 to 71 years old. N. Engl. J. Med..

[3] Boonchaya-anant P., Apovian C.M. (2014). Metabolically healthy obesity—Does it exist?. Curr. Atheroscler. Rep..

[4] Rey-López J.P., de Rezende L.F., Pastor-Valero M., Tess B.H. (2014). The prevalence of metabolically healthy obesity: A systematic review and critical evaluation of the definitions used. Obes. Rev..

[5] Phillips C.M. (2013). Metabolically healthy obesity: Definitions, determinants and clinical implications. Rev. Endocr. Metab. Disord..

[6] Wildman R.P., Muntner P., Reynolds K., McGinn A.P., Rajpathak S., Wylie-Rosett J., Sowers M.R. (2008). The obese without cardiometabolic risk factor clustering and the normal weight with cardiometabolic risk factor clustering: Prevalence and correlates of 2 phenotypes among the US population (NHANES 1999–2004). Arch. Intern. Med..

[7] Bell J.A., Kivimaki M., Hamer M. (2014). Metabolically healthy obesity and risk of incident type 2 diabetes: A meta-analysis of prospective cohort studies. Obes. Rev..

[8] Kramer C.K., Zinman B., Retnakaran R. (2013). Are metabolically healthy overweight and obesity benign conditions? A systematic review and meta-analysis. Ann. Intern. Med..

[9] Hankinson A.L., Daviglus M.L., van Horn L., Chan Q., Brown I., Holmes E., Elliott P., Stamler J. (2013). Diet composition and activity level of at risk and metabolically healthy obese American adults. Obesity (Silver Spring).

[10] Lee K. (2009). Metabolically obese but normal weight (MONW) and metabolically healthy but obese (MHO) phenotypes in Koreans: Characteristics and health behaviors. Asia Pac. J. Clin. Nutr..

[11] Phillips C.M., Dillon C., Harrington J.M., McCarthy V.J., Kearney P.M., Fitzgerald A.P., Perry I.J. (2013). Defining metabolically healthy obesity: Role of dietary and lifestyle factors. PLoS ONE.

[12] Wang Y., Mi J., Shan X.Y., Wang Q.J., Ge K.Y. (2007). Is China facing an obesity epidemic and the consequences? The trends in obesity and chronic disease in China. Int. J. Obes. (Lond.).

[13] Bassett D.R. (2003). International physical activity questionnaire: 12-country reliability and validity. Med. Sci. Sports Exerc..

[14] Zhou B.F. (2002). Cooperative Meta-analysis group of the working group on obesity in C. Predictive values of body mass index and waist circumference for risk factors of certain related diseases in Chinese adults—Study on optimal cut-off points of body mass index and waist circumference in Chinese adults. Biomed. Environ. Sci..

[15] Alberti K.G., Zimmet P., Shaw J. (2005). IDF Epidemiology task force consensus group. The metabolic syndrome—A new worldwide definition. Lancet.

[16] Kuk J.L., Ardern C.I. (2009). Are metabolically normal but obese individuals at lower risk for all-cause mortality?. Diabetes Care.

[17] Voulgari C., Tentolouris N., Dilaveris P., Tousoulis D., Katsilambros N., Stefanadis C. (2011). Increased heart failure risk in normal-weight people with metabolic syndrome compared with metabolically healthy obese individuals. J. Am. Coll. Cardiol..

[18] Iacobellis G., Ribaudo M.C., Zappaterreno A., Iannucci C.V., Leonetti F. (2005). Prevalence of uncomplicated obesity in an Italian obese population. Obes. Res..

[19] Heianza Y., Arase Y., Tsuji H., Fujihara K., Saito K., Hsieh S.D., Tanaka S., Kodama S., Hara S., Sone H. (2014). Metabolically healthy obesity, presence or absence of fatty liver, and risk of type 2 diabetes in Japanese individuals: Toranomon hospital health management center study 20 (TOPICS 20). J. Clin. Endocrinol. Metab..

[20] Geetha L., Deepa M., Anjana R.M., Mohan V. (2011). Prevalence and clinical profile of metabolic obesity and phenotypic obesity in Asian Indians. J. Diabetes Sci. Technol..

[21] Skadhauge L.R., Christensen K., Kyvik K.O., Sigsgaard T. (1999). Genetic and environmental influence on asthma: A population-based study of 11,688 Danish twin pairs. Eur. Respir. J..

[22] Otsuki M., Kasayama S., Morita S., Asanuma N., Saito H., Mukai M., Koga M. (2007). Menopause, but not age, is an independent risk factor for fasting plasma glucose levels in nondiabetic women. Menopause.

[23] Gu D., Reynolds K., Wu X., Chen J., Duan X., Reynolds R.F., Whelton P.K., He J. (2005). InterASIA Collaborative Group. Prevalence of the metabolic syndrome and overweight among adults in China. Lancet.

[24] Sung K.C., Cha S.C., Sung J.W., So M.S., Byrne C.D. (2014). Metabolically healthy obese subjects are at risk of fatty liver but not of pre-clinical atherosclerosis. Nutr. Metab. Cardiovasc. Dis..

[25] Bays H.E. (2011). Adiposopathy is “sick fat” a cardiovascular disease?. J. Am. Coll. Cardiol..

[26] Shah R.V., Murthy V.L., Abbasi S.A., Blankstein R., Kwong R.Y., Goldfine A.B., Jerosch-Herold M., Lima J.A., Ding J., Allison M.A. (2014). Visceral adiposity and the risk of metabolic syndrome across body mass index: The MESA Study. JACC Cardiovasc. Imaging..

[27] Primeau V., Coderre L., Karelis A.D., Brochu M., Lavoie M.E., Messier V., Sladek R., Rabasa-Lhoret R. (2011). Characterizing the profile of obese patients who are metabolically healthy. Int. J. Obes..

[28] Katzmarzyk P.T., Church T.S., Blair S.N. (2004). Cardiorespiratory fitness attenuates the effects of the metabolic syndrome on all-cause and cardiovascular disease mortality in men. Arch. Intern. Med..

[29] Li T.Y., Rana J.S., Manson J.E., Willett W.C., Stampfer M.J., Colditz G.A., Rexrode K.M., Hu F.B. (2006). Obesity as compared with physical activity in predicting risk of coronary heart disease in women. Circulation.

[30] Conus F., Allison D.B., Rabasa-Lhoret R., St-Onge M., St-Pierre D.H., Tremblay-Lebeau A., Poehlman E.T. (2004). Metabolic and behavioral characteristics of metabolically obese but normal-weight women. J. Clin. Endocrinol. Metab..

[31] Visioli F. (2011). Nutritional support in the pharmacological treatment of metabolic syndrome. Eur. J. Pharmacol..

[32] Lee Y., Kang D., Lee S.A. (2014). Effect of dietary patterns on serum C-reactive protein level. Nutr. Metab. Cardiovasc. Dis..

[33] Sangun Ö., Dündar B., Köşker M., Pirgon Ö., Dündar N. (2011). Prevalence of metabolic syndrome in obese children and adolescents using three different criteria and evaluation of risk factors. J. Clin. Res. Pediatr. Endocrinol..

[34] Singh R., Martin B.R., Hickey Y., Teegarden D., Campbell W.W., Craig B.A., Schoeller D.A., Kerr D.A., Weaver C.M. (2009). Comparison of self-reported, measured, metabolizable energy intake with total energy expenditure in overweight teens. Am. J. Clin. Nutr..

